# Draft Whole-Genome Sequence and Annotation of *Xenorhabdus griffiniae* Strain BMMCB Associated with the South African Entomopathogenic Nematode *Steinernema khoisanae* Strain BMMCB

**DOI:** 10.1128/genomeA.00785-15

**Published:** 2015-07-16

**Authors:** Boipelo Mothupi, Jonathan Featherston, Vincent Gray

**Affiliations:** a School of Molecular and Cell Biology, University of the Witwatersrand, Johannesburg, South Africa; b Agricultural Research Council, Biotechnology Platform, Pretoria, South Africa

## Abstract

*Xenorhabdus griffiniae* strain BMMCB (LDNM00000000) belongs to the family *Enterobacteriaceae* and was isolated from the South African entomopathogenic nematode *Steinernema khoisanae* strain BMMCB (GenBank accession no. KT027382). Here, we report the draft whole-genome sequence of *X. griffinae* strain BMMCB with a genome size of 4,183,779 bp and 44.7% G+C content. The NCBI Prokaryotic Automatic Annotation Pipeline (PGAAP) revealed 3,970 genes.

## GENOME ANNOUNCEMENT

*Xenorhabdus* species are symbiotic Gram-negative bacteria which belong to the *Enterobacteriaceae* family and have a specific obligatory association with *Steinernematid* entomopathogenic nematodes (EPNs) (1). The EPN-bacteria complex holds great potential as biocontrol agents of insect pests (2). The *Steinernematid* EPN infective juvenile (IJ) gains entry through the natural openings of insect host larvae and regurgitate the *Xenorhabdus* bacterial endosymbiont into the hemolymph (1). Following infection, *Xenorhabdus* spp. depress the insect host’s immune system and release insecticidal metabolites, which are lethal to the insect (3). Bactericidal, fungicidal, and nematicidal compounds released by the bacteria facilitate the maintenance of a monoxenic environment within the infected host’s hemocoel (4–6). Several compounds with antibiotic activity secreted by *Xenorhabdus* spp. include benzylineacetone (7), xenorhabdins and xenocoumacin (8), phenethylamides (9), and cyclolipopeptide (10). In this study, we present the description of the *X. griffiniae* BMMCB draft whole-genome sequence and annotation.

*X. griffiniae* BMMCB was isolated from the hemolymph of *Galleria mellonella* larvae infected with the South African entomopathogenic nematode *Steinernema khoisanae* BMMCB (isolation methods are described in reference 11). Genomic DNA was extracted from colony bacterial cultures using the ZR Fungal/Bacteria DNA kit (catalogue D6005) and was purified with the DNA clean and concentrator −5 kit (catalogue D4013). The strain was identified through the amplification and sequencing of the 16S rDNA. On NCBI BLAST, the results showed a 98% similarity percentage to *Xenorhabdus griffiniae* and was named *Xenorhabdus griffiniae* strain BMMCB.

Illumina libraries were generated using the Illumina Nextera DNA sample preparation kit (FC-121-1031) and sequenced using an Illumina MiSeq instrument (version 3, chemistry 300 × 300 bp). CLC Genomic Workbench v7 (CLC bio) was used for quality trimming of adapter sequences and merging of overlapping paired reads. The *X. griffiniae* BMMCB genome was subsequently assembled *de novo* and 231 contigs (≥400 bp) were generated with an average coverage of 232×. The whole-genome sequence comprises 4,183,779 bp, with an *N*_50_ of 57,901 bp and G+C content of 44.7%, similar to those of other *Xenorhabdus* spp. (10). The assembly was submitted for annotation to the NCBI Prokaryotic Genome Automatic Annotation Pipeline (PGAAP) server (http://www.ncbi.nlm.nih.gov/genome/annotation_prok/). Annotation revealed a total of 3,970 genes (3,614 protein coding [CDs] and 271 pseudogenes). The genome has 12 rRNAs (5S, 16S, and 23S), 70 tRNAs, and 3 noncoding RNAs. The polyketide synthase genes involved in biosynthetic pathway of antibiotic compounds have been identified.

### Nucleotide sequence accession numbers. 

This whole-genome shotgun project has been deposited at DDBJ/EMBL/GenBank under the accession no. LDNM00000000. The version described in this paper is LDNM01000000.
